# Sulforaphane ameliorates lipid profile in rodents: an updated systematic review and meta-analysis

**DOI:** 10.1038/s41598-021-87367-9

**Published:** 2021-04-08

**Authors:** Kaili Du, Yuxin Fan, Dan Li

**Affiliations:** Collaborative Innovation Center of Yangtze River Delta Region Green Pharmaceuticals, College of Pharmaceutical Sciences, Zhejiang University of Technology, Hangzhou, China

**Keywords:** Computational biology and bioinformatics, Data integration, Data publication and archiving

## Abstract

Sulforaphane (SFN), a naturally-occurring isothiocyanate enriched in cabbage and broccoli, has been provided as food supplements to improve weight management and reduce lipid levels. However, its effects on serum lipid profiles are contradictory. In this review, a meta-analysis and systematic review of SFN on lipid reduction and weight control is assessed with mice and rats fed on high-fat diet. The effects of SFN supplementation were evaluated by weighted mean difference (WMD) in body weight (BW), liver weight (LW) and also by its effect on serum lipids. A random-effects model was applied to estimate the overall summary effect. SFN reduced BW (WMD: − 2.76 g, 95% CI: − 4.19, − 1.34) and LW (WMD: − 0.93 g, 95% CI: − 1.63, − 0.23) significantly in our ten trials. Its effects on serum total cholesterol (TC) (WMD: − 15.62 mg/dL, 95% CI: − 24.07, − 7.18), low-density lipoprotein cholesterol (LDL-C) (WMD: − 8.35 mg/dL, 95% CI: − 15.47, − 1.24) and triglyceride (TG) (WMD: − 40.85 mg/dL, 95% CI: − 67.46, − 14.24) were significant except for high-density lipoprotein cholesterol (HDL-C) component (WMD: 1.05 mg/dL, 95% CI: − 3.44, 5.54). However, species, disease model, duration, SFN dosage as well as route of administration did not explain the heterogeneity among studies. In summary, these findings provide new insights concerning preclinical strategies for treating diseases including obesity, diabetes, hypertension, non-alcoholic fatty liver disease as well as cardiovascular disease with SFN supplements.

## Introduction

Chronic, non-communicable diseases and metabolic syndromes including obesity, diabetes, hypertension, hypercholesterolaemia, cardiovascular disease (CVD) and non-alcoholic fatty liver disease (NAFLD)^1–3^ are significantly increasing due to lifestyle and dietary patterns. This coincides with modifications in global nutritional and epidemiological characteristics^4^. Lipid profile as well as obesity are risk factors for CVD^5^. Therefore, uptake of a balanced diet and natural product supplements has been recommended to reduce the risk of metabolic syndrome through weight management and lipid reduction to normal physiological levels^6,7^.

In the past decades, natural products derived from plants are utilized to prevent or treat obesity, diabetes and lipids-associated disorders^8–10^. Epidemiological studies suggest that supplementation with plant-derived bioactive compounds can be beneficial in control of body weight and reduction of lipid accumulation^11^. Sulforaphane (SFN), a natural product enriched in broccoli and cabbage, can decrease lipid levels both in vivo and in vitro^6,12,13^. These studies suggest SFN may become a potential therapeutic drug for dyslipidemia. SFN has also been effective in the treatment of atherosclerosis, diabetes, and neurodegenerative diseases^14–16^.

Two randomized clinical trials (RCTs) show that intake of broccoli sprouts can significantly reduce inflammatory markers and plasma low-density lipoprotein cholesterol (LDL-C) in the long term^17,18^. Animal studies also show that SFN upregulates the expression of phase I & phase II metabolic enzymes and lipid metabolism-related enzymes/proteins^19^. Moreover, SFN induces adipocyte lipolysis and inhibits adipocyte differentiation^20–23^, which could be the possible mechanism that SFN improves lipid profile.

So far only two RCTs of serum lipid profile on SFN are reported. Thus, in this study, we focus on the effect of SFN on lipid regulation in the preclinical studies (rats and mice)^17,18^. Particularly, no meta-analysis about mono-treatment of SFN on lipid instead of using food enriched with SFN. Therefore, we here, provide a systematic review and meta-analysis that summarizes SFN effects on lipid profiles in animals since 2013.

## Material and method

### Search strategy

To find articles related to SFN, we systematically searched databases including: Web of Science, PubMed, SCOPUS, medRxiv, bioRxiv and Google Scholar from 2013 through Sept. 2020. Our search of those databases utilized MeSH and non-MeSH terms related to lipid profile and sulforaphane. These included: “Lipoproteins, LDL”, “Low Density Lipoprotein Cholesterol”, “Cholesterol LDL”, “LDL triacylglycerol”, “Triglycerides”, “Triacylglycerol”, “Triacylglycerols”, “Lipoproteins, HDL”, “HDL Lipoproteins”, “High Density Lipoproteins”, “High-Density Lipoproteins”, “Lipoproteins, High-Density”, “Lipoproteins, VLDL”, “Cholesterol”, “Cholesterol, VLDL”, “VLDL Cholesterol”, “Very Low Density Lipoprotein Cholesterol”, “Very-Low-Density Lipoproteins”, “Lipoproteins, Very-Low-Density”, “Very Low Density Lipoproteins”, “Lipoproteins VLDL”, “VLDL Lipoproteins”, “Lipoproteins, VLDL”, “total cholesterol”, “TC”, “LDL”, “HDL”, “VLDL”, “TG”, “Lipolysis” and “sulforaphane”^24^. Initially, we attempted to focus our study on the clinical trials of SFN. Regrettably, only two clinical research relevant to the lipid profile of SFN are reported. The two research also lack some key clinical indicators including dosage of drug, duration of treatment and route of administration. Hence, we decided to concentrate on the preclinical studies of SFN (rodents-rats and mice).

### Inclusion criteria

This systematic review and meta-analysis was conducted according to the PRISMA guidelines^25^. Studies with following criteria were selected for meta-analysis: (i) Original articles; (ii) Focusing on rodent (rats and mice) models; (iii) Using SFN as monotherapy in intervention group; (iv) Evaluation of systemic metabolic parameters, including body weight (BW), liver weight (LW) or lipid profile data.

### Exclusion criteria

We excluded trials if they met the following criteria: (A) Using food sources instead of SFN, (B) Using other food supplements with SFN, (C) Lacking of control group, (D) Having unclear/inadequate data, (E) Not using rodents (rats and mice) model, (F) Acute SFN action.

### Data extraction

After considering criteria of inclusion and exclusion, eligible articles were selected. Detailed data includes: the name of the first author, publication year, species and sex, number of animals, age of animals, model method, duration, intervention (including SFN dose, route of administration), the main outcomes, intergroup difference and results^26^.

### Statistical analysis

Treatment effects were considered as weighted mean difference (WMD) and the corresponding standard error (SE) in BW, LW and concentrations of serum lipids (TC, LDL-C, HDL-C and TG). To estimate the overall effect, we used a random-effect model, previously described by DerSimonian and Laird, which considers both within and between-study heterogeneity^27^. Heterogeneity among the studies was estimated using the I^2^ statistic, with values of 0–25%, 25.1–75%, and 75.1–100% representing a low, moderate, and high degree of heterogeneity, respectively. When standard deviations or SEs were not shown in studies, they were calculated using 95% CI. In addition, when studies have reported median and interquartile range, they were converted to mean and SE using available formulas^28^. Statistical analyses were done using Stata, version 13 (Stata Corp., College Station, TX, USA). P-values less than 0.05 were considered statistically significant.

## Results

### Selection of articles

**Figure 1:**
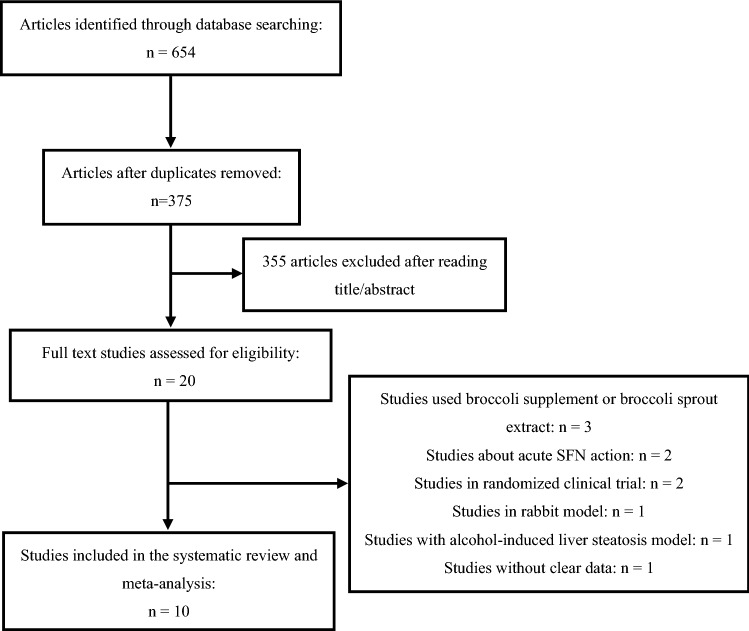
Flow diagram of database searches and study selection.

A total of 654 studies were involved by our database search. 279 duplicate articles were removed. After reading the title and abstract of papers, 20 articles were selected to analyze the full text with removing 355 studies. We considered inclusion and exclusion criteria and then excluded 10 further studies owing to prescribing broccoli supplement or broccoli sprout extract instead of SFN (n = 3), acute SFN action (n = 2), RCTs (n = 2), rabbit model (n = 1), alcohol-induced liver steatosis model (n = 1) and lacking of clear data (n = 1) (Fig. 1). Ultimately, this meta-analysis was conducted on ten trials of rodents^29–38^ (Table 1), including 5 batches of C57BL/6 mice, 4 batches of Wistar rats and 1 batch of Sprague Dawley rats. In terms of gender, all the trials selected male animals aged 4–10 weeks. The methods to build disease models included feeding mice or rats with high-fat diet, high-fructose diet or highly palatable diet or injecting of streptozotocin (STZ) into rodents. Specifically, four trials induced obesity by feeding with high-fat diet, one trial by feeding with high-fructose diet, one trial by feeding with high-fat high-sucrose diet, and one trial by feeding with highly palatable diet, two trials evoked diabetes by feeding with high-fat diet and then injecting of STZ and one trail by injection of STZ. The animals were treated with SFN using multiple routes, including by oral administration, oral gavage, subcutaneous injection, and intraperitoneal injection. The intervention duration was 3 to 16 weeks. The dosage of SFN ranged from 0.5 mg/kg to 30 mg/kg.

**Table 1:** Description of included studies.

First author, year	Species	No. of animals (intervention/control group)	Age (weight)	Model method (time)	Duration	Intervention	The main outcomes	Intergroup difference	Results
1. Choi K.M. (2014)^29^	Male C57BL/6N mice	10/10	4 weeks	High-fat diet (6 weeks)	6 weeks	1 g/kg diet SFN, oral administration	1. BW 2. TC (serum) 3. TG (serum) 4. HDL-C 5. LDL-C 6. TC (liver) 7. TG (liver)	1. P < 0.01 2. P < 0.05 3. P > 0.05 4. P > 0.05 5. P > 0.05 6. P > 0.05 7. P > 0.05	BW and serum TC, decreased significantly, but serum TG, HDL-C, LDL-C, liver TC, and TG did not change in SFN group
2. Lei P (2019)^52^	Male Wistar rats	6/6	4–6 weeks (160–200 g)	High-fat diet (10 weeks)	10 weeks	20 mg/kg, 3 days a week SFN, oral gavage	1. LW 2. TC (serum) 3. TG (serum) 4. HDL-C 5. LDL-C 6. TC (liver) 7. TG (liver) 8. LDs (liver) 9. FFA (liver)	1. P < 0.05 2. P < 0.05 3. P < 0.05 4. P > 0.05 5. P < 0.05 6. P > 0.05 7. P < 0.05 8. P < 0.01 9. P < 0.01	LW_,_ serum TC, TG, LDL-C, liver TG and LDs decreased significantly, but serum HDL-C, and liver TC did not change in SFN group
3. Shawky N.M. (2019)^31^	Male Sprague Dawley rats	8/10	8 weeks (150–200 g)	High-fructose diet (9 weeks)	6 weeks	0.5 mg/kg/day SFN, oral gavage	1. BW 2. AUC_OGTT_ 3. AUC_ITT_ 4. HOMA-IR 5. TC (serum) 6. TG (serum) 7. HDL-C 8. LDL-C	1. P > 0.05 2. P < 0.05 3. P > 0.05 4. P < 0.05 5. P > 0.05 6. P > 0.05 7. P < 0.05 8. P < 0.05	AUC_OGTT,_ HOMA-IR, serum HDL-C and LDL-C ameliorated significantly, but BW, AUC_ITT_, serum TC and TG did not change in SFN group
4. Shawky N.M. (2016)^32^	Male C57BL/6J mice	11/11	8 weeks	High-fat high-sucrose diet (8 weeks)	3 weeks	0.5 mg/kg/day SFN, subcutaneous injection	1. BW 2. HOMA-IR 3. AUC_IPGTT_ 4. TC (plasma) 5. TG (plasma) 6. HDL-C (plasma) 7. LDL-C (plasma) 8. FFA (plasma) 9. Non-HDL-C (plasma)	1. P < 0.05 2. P < 0.05 3. P < 0.05 4. P > 0.05 5. P < 0.05 6. P < 0.05 7. P > 0.05 8. P < 0.05 9. P < 0.05	BW, HOMA-IR, AUC_IPGTT,_ plasma TG, HDL-C, FFA and non-HDL-C ameliorated significantly, but plasma TC, LDL-C did not change in SFN group
5. Souza C.G. (2016)^33^	Male Wistar rats	8/7	8 weeks	Injection of STZ	3 weeks	0.5 mg/kg/day SFN, intraperitoneal injection	1. LW 2. TC (serum) 3. HDL-C (serum) 4. Non-HDL-C 5. TG (serum) 6. ALT 7. AST 8. AUC_IPIRT_	1. P > 0.05 2. P < 0.05 3. P > 0.05 4. P < 0.05 5. P < 0.05 6. P > 0.05 7. P > 0.05 8. P < 0.05	Serum TC, non-HDL-C, TG, AUC_IPIRT_ decreased significantly, but LW, HDL-C, ALT, and AST did not change in SFN group
6. Souza C.G. (2013)^34^	Male Wistar rats	7/7	8 weeks	Highly palatable diet (24 weeks) (enriched sucrose diet)	16 weeks	1 mg/kg/day SFN, oral gavage	1. BW 2. LW 3. TC (serum) 4. HDL-C 5. TAG (serum) 6. TAG (liver) 7. TC (liver) 8. ALT 9. AST 10.AUC_IPGTT_	1. P > 0.05 2. P > 0.05 3. P > 0.05 4. P > 0.05 5. P > 0.05 6. P > 0.05 7. P > 0.05 8. P > 0.05 9. P > 0.05 10.P > 0.05	Lipid parameters did not change significantly in SFN group
7. Sun Y. (2020)^35^	Male C57BL/6J mice	5/5	8 weeks	High-fat diet (12 weeks, 24 weeks), injection of STZ	12 weeks	0.5 mg/kg, 5 days a week SFN, intraperitoneal injection	1. BW 2. LDs (cardiac)	1. P > 0.05, P < 0.05 2. P > 0.05, P < 0.05	BW and cardiac LDs did not change significantly in SFN group treated by HFD for 12 weeks, but both of them decreased significantly in SFN group treated by HFD for 24 weeks
8. Tian S. (2017)^36^	Male Wistar rats	10/10	4–6 weeks (160–200 g)	High-fat diet (10 weeks)	10 weeks	5, 10, 20 mg/kg, 3 days a week SFN, oral gavage	1. TC (plasma) 2. TG (plasma) 3. TC (liver) 4. TG (liver)	1. P < 0.05, P < 0.05, P < 0.0001 2. P < 0.0001, P < 0.05, P < 0.0001 3. P > 0.05, P < 0.01, P < 0.0001 4. P > 0.05, P < 0.0001, P < 0.0001	Plasma TC, TG decreased significantly, but liver TC, TG did not change significantly in low-doses-SFN group; All of plasma TC, TG, liver TC and TG decreased significantly in middle-doses-SFN and high-doses-SFN group
9. Yang G. (2016)^37^	Male C57BL/6 mice	8/8	5 weeks	High-fat diet (9 weeks)	9 weeks	30 mg/kg/day SFN, oral gavage	1. LW 2. TC (liver) 3. TG (liver) 4. FFA (liver) 5. ALT 6. AST 7. HOMA-IR	1. P < 0.05 2. P < 0.05 3. P < 0.05 4. P < 0.05 5. P < 0.05 6. P < 0.05 7. P < 0.05	Lipid parameters change significantly in SFN group
10. Zhang Z. (2014)^38^	Male C57BL/6J mice	6/6	8–10 weeks	High-fat diet (12 weeks), injection of STZ	16 weeks	0.5 mg/kg, 5 days a week SFN, subcutaneous injection	1. TC (plasma) 2. TG (plasma) 3. AUC_IPGTT_ 4. LDs (cardiac)	1. P > 0.05 2. P < 0.05 3. P > 0.05 4. P < 0.05	Plasma TG and cardiac LDs decreased significantly, but plasma TC and AUC_IPGTT_ did not change significantly in SFN group

*ALT* alanine aminotransferase, *AST* aspartate aminotransferase, *AUC* area under the curve, *FFA* free fatty acids, *GTT* glucose tolerance test, *HOMA-IR* an index of insulin resistance, *IP* intraperitoneally, *IRT* the insulin responsiveness test, *LDs* lipid droplets, *O* oral.

### Effects of SFN supplementation on body weight

**Figure 2:**
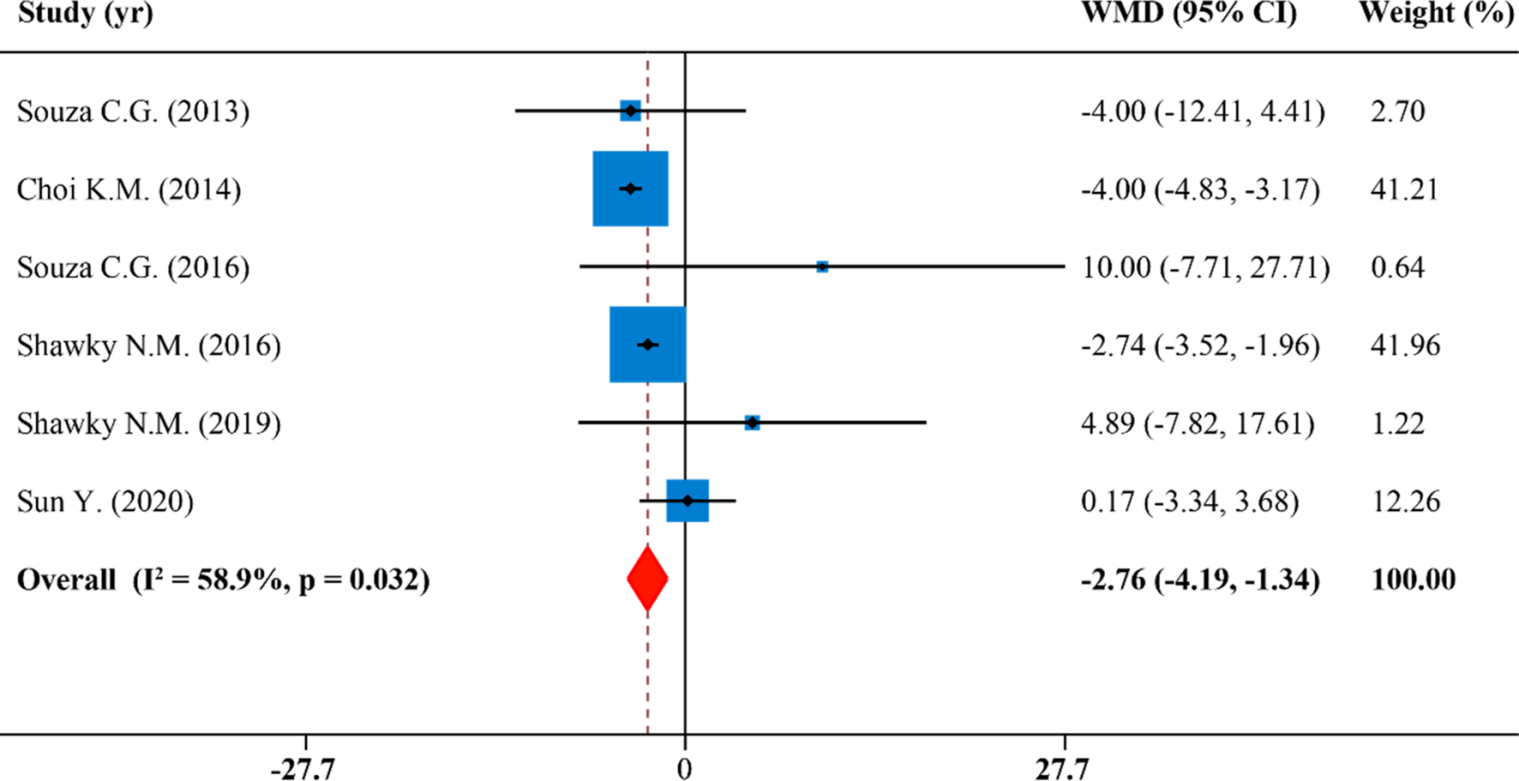
Forest plot showing effects of SFN on body weight.

The meta-analysis of BW included 6 publications with 6 effect sizes. We found that SFN supplementation was correlated with BW changes (WMD: − 2.76 g, 95% CI: − 4.19, − 1.34; P = 0.032, I^2^ = 58.9%) (Fig. 2). In the subgroup analyses, the heterogeneity was not observed when studies were stratified by species, disease model, duration and SFN dosage and administration route (Table 2). However, heterogeneity was attenuated in oral administration subgroup (I^2^ = 0.0%), studies which lasted for > 10 weeks (I^2^ = 0.0%), diabetes model subgroup (I^2^ = 12.2%), studies whose dosage of SFN ≤ 0.5 mg/kg/d (I^2^ = 12.2%) and rats subgroup (I^2^ = 24.8%). SFN supplementation significantly decreased BW in murine group (WMD: − 2.93 g, 95% CI: − 4.34, − 1.52; P = 0.015, I^2^ = 76.3%), in the obesity model studies (WMD: − 3.30 g, 95% CI: − 4.46, − 2.14; P = 0.096, I^2^ = 52.7%), in studies which lasted for ≤ 10 weeks (WMD: − 3.18 g, 95% CI: − 4.60, − 1.75; P = 0.037, I^2^ = 64.6%), in studies whose dosage of SFN > 0.5 mg/kg/d (WMD: − 3.30 g, 95% CI: − 4.46, − 2.14; P = 0.096, I^2^ = 52.7%), in the oral administration group (WMD: − 3.96 g, 95% CI: − 4.47, − 3.14; P = 0.392, I^2^ = 0.0%) and in the injection administration group (WMD: − 1.33 g, 95% CI: − 4.46, 1.80; P = 0.107, I^2^ = 55.2%). However, BW was not reduced in rats group (WMD: 1.12 g, 95% CI: − 6.87, 9.19; P = 0.265, I^2^ = 24.8%) nor the diabetes model of rodents with a dosage of SFN ≤ 0.5 mg/kg/day (WMD: 1.09 g, 95% CI: − 4.53, 6.72; P = 0.286, I^2^ = 12.2%) (Table 2).

**Table 2:** Subgroup analysis to assess the effect of SFN supplement on lipid profile.

	Effect size, n	WMD	95% CI	I-squared (%)	P for heterogeneity	P for between subgroup heterogeneity
**Body weight**						
Overall effect	6	− 2.76	− 4.19, − 1.34	58.9	0.032	
**Species**						0.471
Mice	3	− 2.93	− 4.34, − 1.52	76.3	0.015	
Rats	3	1.12	− 6.87, 9.10	24.8	0.265	
**Disease model**						0.122
Obesity (without STZ)	4	− 3.30	− 4.46, − 2.14	52.7	0.096	
Diabetes (with STZ)	2	1.09	− 4.53, 6.72	12.2	0.286	
**Duration**						0.122
≤ 10 weeks	4	− 3.18	− 4.60, − 1.75	64.6	0.037	
> 10 weeks	2	− 0.45	− 3.68, 2.79	0.0	0.37	
**SFN dosage**						0.292
≤ 0.5 mg/kg/day	2	1.09	− 4.53, 6.72	12.2	0.286	
> 0.5 mg/kg/day	4	− 3.30	− 4.46, − 2.14	52.7	0.096	
**Administration route**						0.391
Oral	3	− 3.96	− 4.47, − 3.14	0.0	0.392	
Injection	3	− 1.33	− 4.46, 1.80	55.2	0.107	
**Liver weight**						
Overall effect	4	− 0.93	− 1.63, − 0.23	93.0	0.000	
**Species**						0.712
Mice	1	− 0.54	− 0.75, − 0.33	–	–	
Rats	3	− 1.11	− 2.31, 0.09	95.2	0.000	
**Disease model**						0.363
Obesity (without STZ)	3	− 1.26	− 2.13, − 0.39	93.3	0.000	
Diabetes (with STZ)	1	0.00	− 0.37, 0.37	–	–	
**Duration**						0.992
≤ 10 weeks	3	− 0.93	− 1.89, 0.03	94.9	0.000	
> 10 weeks	1	− 1.00	− 1.40, − 0.60	–	–	
**SFN dosage**						0.363
≤ 0.5 mg/kg/day	1	0.00	− 0.37, 0.37	–	–	
> 0.5 mg/kg/day	3	− 1.26	− 2.13, − 0.39	93.3	0.000	
**Administration route**						0.363
Oral	3	− 1.26	− 2.13, − 0.39	93.3	0.000	
Injection	1	0.00	− 1.63, − 0.23	–	–	
**Total cholesterol**						
Overall effect	6	− 15.62	− 24.07, − 7.18	92.3	0.000	
**Species**						0.511
Mice	2	− 23.59	− 60.48, 13.30	97.3	0.000	
Rats	4	− 12.77	− 21.41, − 4.14	89.2	0.000	
**Disease model**						0.752
Obesity (without STZ)	4	− 17.49	− 30.34, − 4.64	93.7	0.000	
Diabetes (with STZ)	2	− 12.92	− 28.45, 2.61	94.3	0.000	
**Duration**						0.261
≤ 10 weeks	4	− 21.27	− 34.67, − 7.87	92.5	0.000	
> 10 weeks	2	− 6.17	− 9.01, − 3.34	0.0	0.525	
**SFN dosage**						0.338
≤ 0.5 mg/kg/day	3	− 9.66	− 20.10, 0.78	90.6	0.000	
> 0.5 mg/kg/day	3	− 22.75	− 40.46, − 5.04	95.1	0.000	
**Administration route**						0.752
Oral	4	− 17.49	− 30.34, − 4.64	93.7	0.000	
Injection	2	− 12.92	− 28.45, 2.61	94.3	0.000	
**HDL-C**						
Overall effect	5	1.05	− 3.44, 5.54	91.2	0.000	
**Species**						0.199
Mice	1	− 8.30	− 11.94, − 4.66	–	–	
Rats	4	3.31	− 1.18, 7.80	88.0	0.000	
**Disease model**						0.747
Obesity (without STZ)	4	0.40	− 5.42, 6.22	92.5	0.000	
Diabetes (with STZ)	1	4.00	0.65, 7.35	–	–	
**Duration**						0.878
≤ 10 weeks	4	1.63	− 6.44, 9.70	93.4	0.000	
> 10 weeks	1	0.00	− 0.40, 0.40	–	–	
**SFN dosage**						0.102
≤ 0.5 mg/kg/day	2	8.26	− 0.71, 17.22	85.9	0.008	
> 0.5 mg/kg/day	3	− 3.13	− 8.38, 2.12	90.1	0.000	
**Administration route**						0.747
Oral	4	0.40	− 5.42, 6.22	92.5	0.000	
Injection	1	4.00	0.65, 7.35	–	–	
**Triglyceride**						
Overall effect	5	− 40.85	− 67.46, − 14.24	97.1	0.000	
**Species**						0.659
Mice	2	− 41.49	− 161.29, 78.30	98.7	0.000	
Rats	3	− 33.14	− 60.77, − 5.51	95.3	0.000	
**Disease model**						0.092
Obesity (without STZ)	3	− 1.34	− 18.69, 16.01	95.1	0.000	
Diabetes (with STZ)	2	− 212.55	− 436.50, 11.40	97.1	0.000	
**Duration**						0.898
≤ 10 weeks	4	− 18.05	− 42.06, 5.96	96.4	0.000	
> 10 weeks	1	− 103.25	− 128.94, − 77.56	–	–	
**SFN dosage**						0.305
≤ 0.5 mg/kg/day	3	− 132.41	− 231.71, − 33.12	97.5	0.000	
> 0.5 mg/kg/day	2	4.85	− 22.42, 32.13	97.1	0.000	
**Administration route**						0.092
Oral	3	− 1.34	− 18.69, 16.01	95.1	0.000	
Injection	2	− 212.55	− 436.50, 11.40	94.9	0.000	

### Effects of SFN supplementation on liver weight

**Figure 3:**
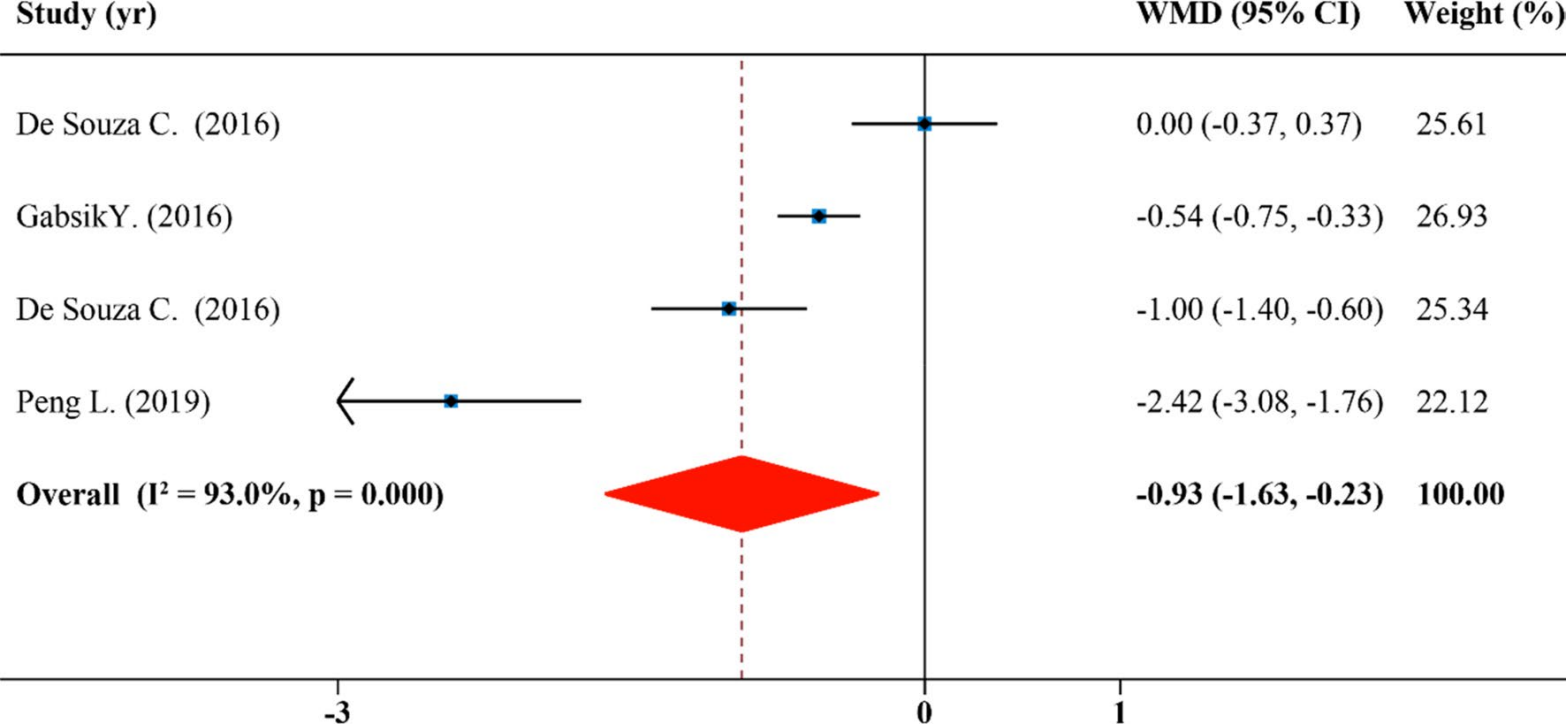
Forest plot showing effects of SFN on liver weight.

The results of LW were calculated in 4 comparisons from 4 studies. As shown in Fig. 3, SFN significantly affected LW of rodents (WMD: − 0.93 g, 95% CI: − 1.63, − 0.23; P = 0.000, I^2^ = 93.0%). In subgroup analyses by the species, disease model, duration, SFN dosage and administration route of rodents, it showed that SFN caused a reduction in levels of LW in the obesity group treated with dosage of SFN > 0.5 mg/kg/day by oral administration (WMD: − 1.26 g, 95% CI: − 2.31, − 0.39; P = 0.000, I^2^ = 95.2%), but its effect in diabetes group treated with dosage of SFN ≤ 0.5 mg/kg/day by injection (WMD: 0.00 g, 95% CI: − 0.37, 0.37; P = 0.000, I^2^ = 95.2%) was not significant (Table 2). Results revealed that classifying trails based on species, disease model, duration, SFN dosage as well as administration route could not explain the heterogeneity among studies from the subgroup analysis (Table 2).

### Effects of SFN supplementation on serum total cholesterol

**Figure 4:**
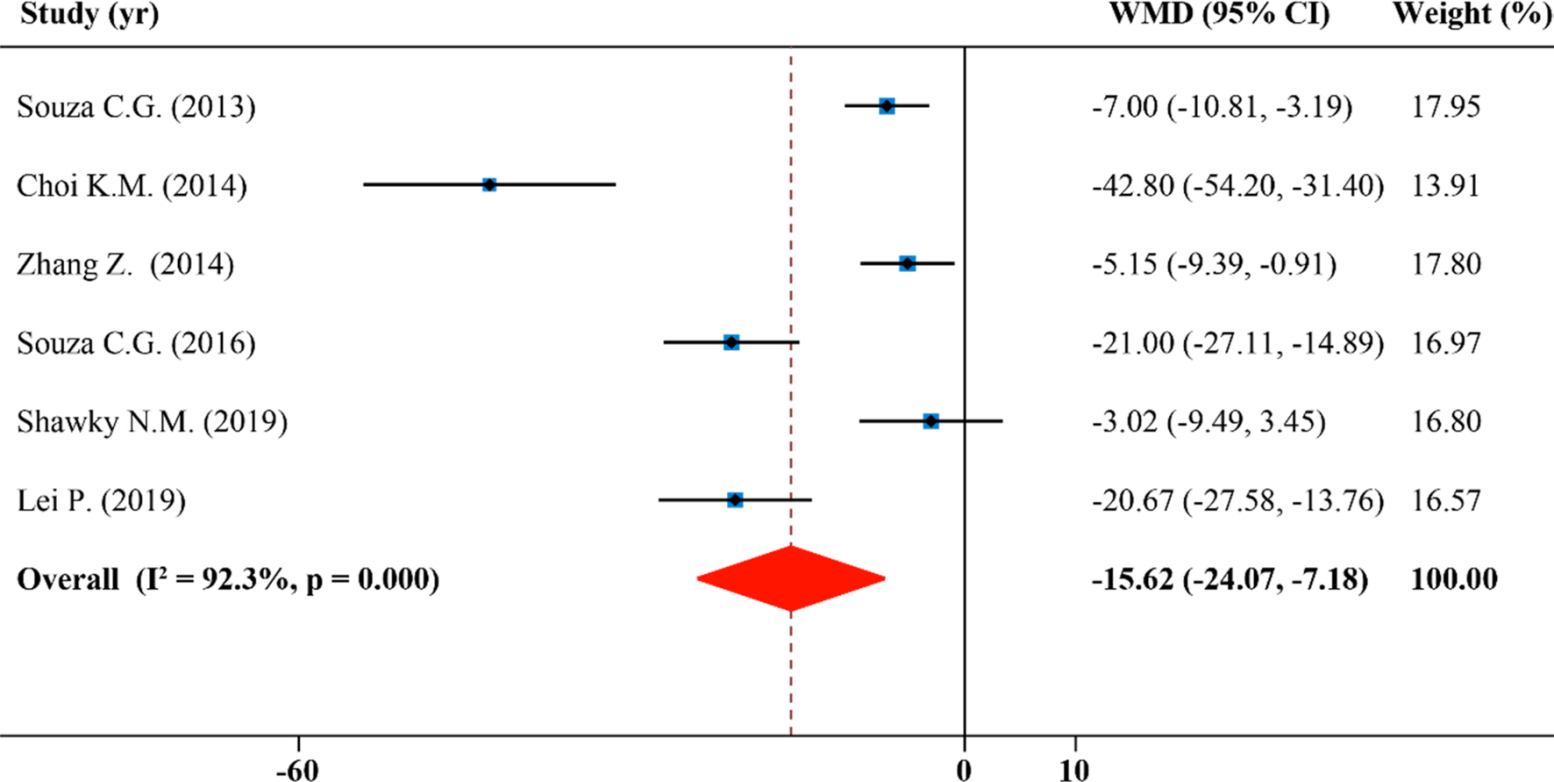
Forest plot showing effects of SFN on serum total cholesterol.

In total 6 publications with 6 effect sizes, serum total cholesterol concentrations were analyzed and reported. SFN caused a significant reduction in serum TC levels (WMD: − 15.62 mg/dL, 95% CI: − 24.07, − 7.18; P = 0.000, I^2^ = 92.3%) (Fig. 4). Heterogeneity was eliminated in studies which lasted for > 10 weeks (I^2^ = 0.0%), however, the heterogeneity sources were not found when studies were stratified duration and other subgroups (Table 2). Moreover, in all studies, intake of SFN led to a significant decline in serum levels of total cholesterol, particularly in the subgroups with a dosage of SFN > 0.5 mg/kg/day (WMD: − 22.75 mg/dL, 95% CI: − 40.46, − 5.04; P = 0.000, I^2^ = 95.1%) and intervention ≤ 10 weeks (WMD: − 21.27 mg/dL, 95% CI: − 34.67, − 7.87; P = 0.000, I^2^ = 92.5%) (Table 2).

### Effects of SFN supplementation on serum low-density lipoprotein cholesterol

**Figure 5:**
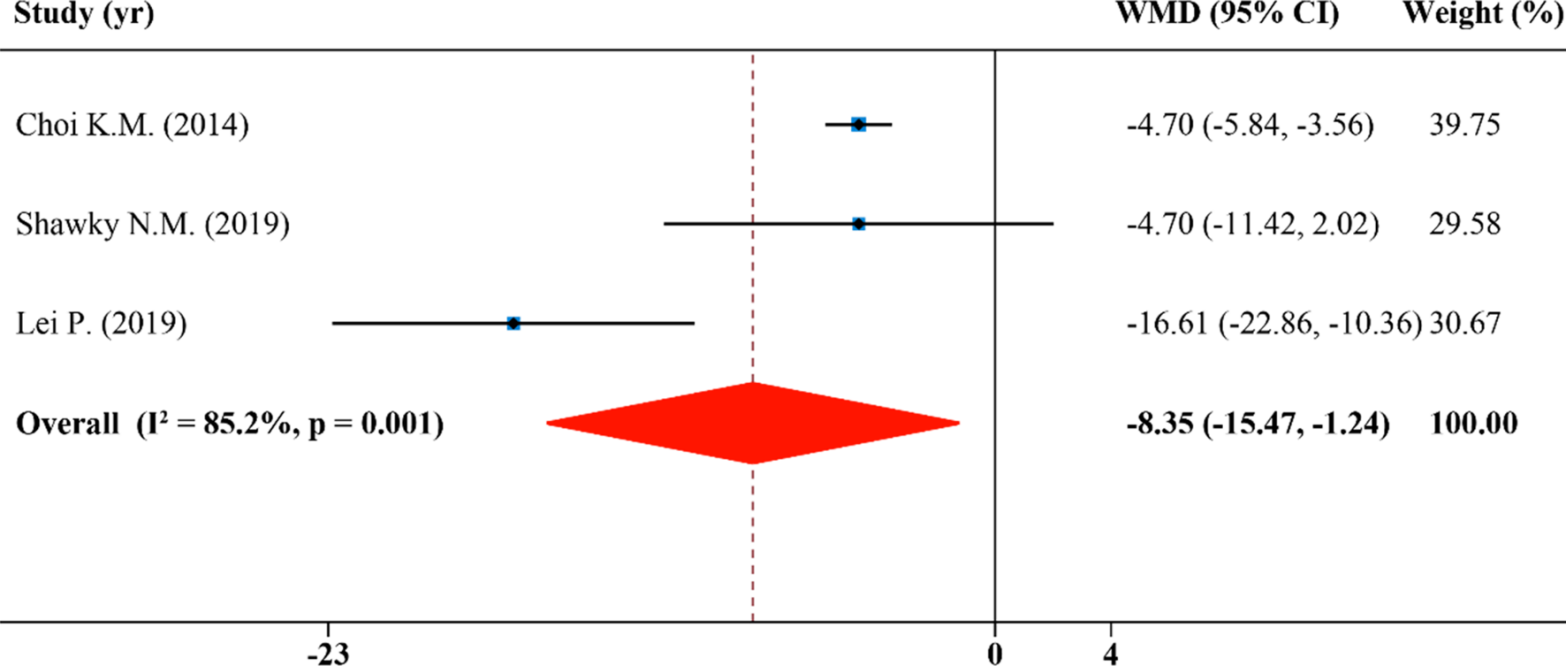
Forest plot showing effects of SFN on serum low-density lipoprotein cholesterol.

In total, the analysis of LDL-C involves 3 publications with 3 effect sizes. A statistically significant reduction effect of SFN supplementation on serum LDL-C (WMD: − 8.35 mg/dL, 95% CI: − 15.47, − 1.24; P = 0.001, I^2^ = 85.2%) was discovered (Fig. 5).

### Effects of SFN supplementation on serum high-density lipoprotein cholesterol

**Figure 6:**
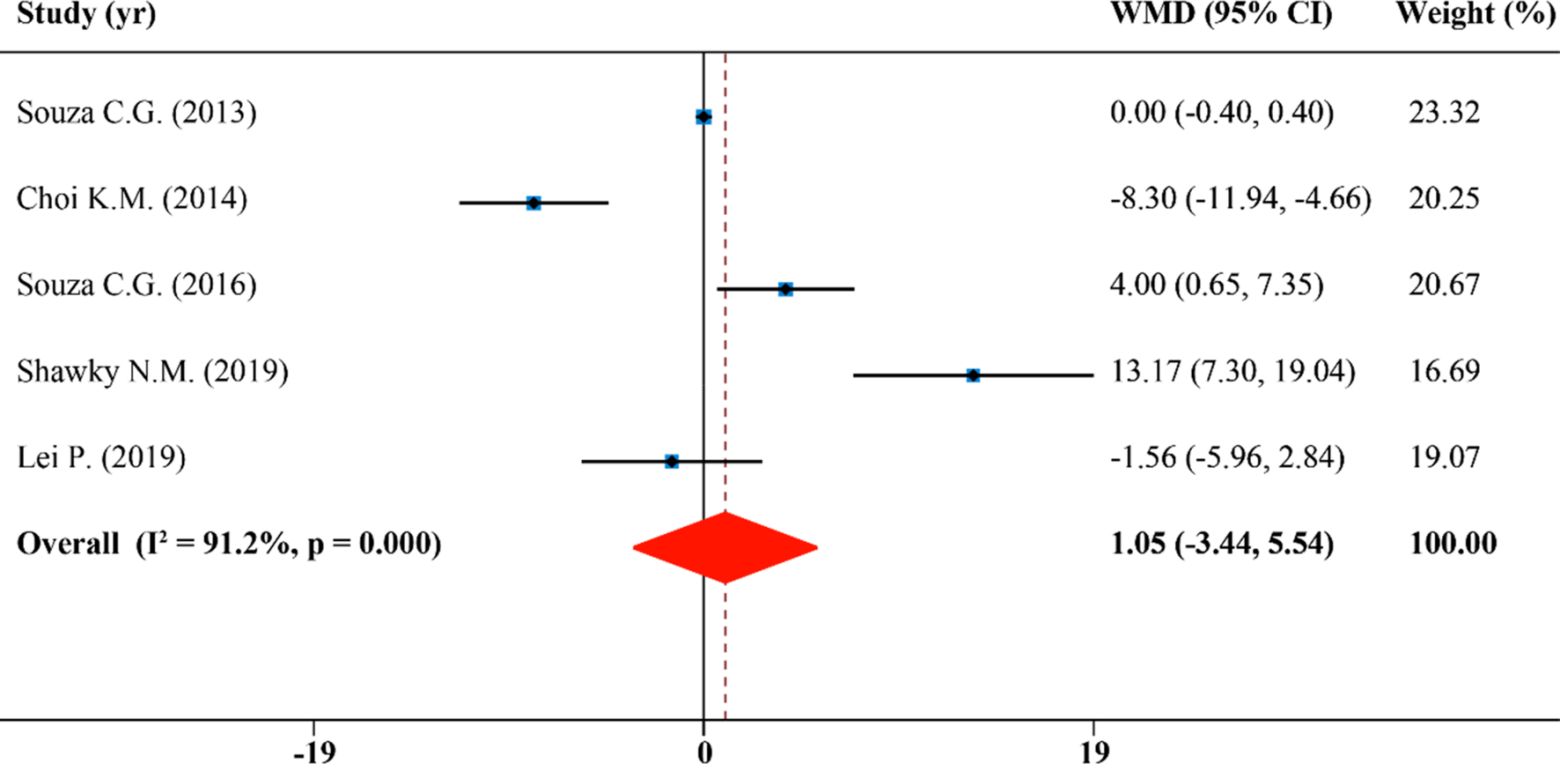
Forest plot showing effects of SFN on serum high-density lipoprotein cholesterol.

Combining 5 effect sizes from 5 publications, we found SFN was not effective in raising the levels of serum HDL-C concentration (WMD: 1.05 mg/dL, 95% CI: − 3.44, 5.54; P = 0.000, I^2^ = 91.2%) (Fig. 6). In subgroup analyses, it appears that SFN played a role in a significant increase of HDL-C in diabetes subgroup which is injected with SFN (WMD: 4.00 mg/dL, 95% CI: 0.65, 7.35)^33^, but it had no significant effect in the obesity group by oral administration (WMD: 0.40 mg/dL, 95% CI: − 5.42, 6.22; P = 0.000, I^2^ = 92.5%). In addition, SFN supplementation reduced the level of HDL-C significantly in the murine subgroup (WMD: − 8.30 mg/dL, 95% CI: − 11.94, − 4.66). Species, disease model, duration and SFN dosage and administration route of studies were not determined to be sources of heterogeneity (Table 2).

### Effects of SFN supplementation on serum triglyceride

**Figure 7:**
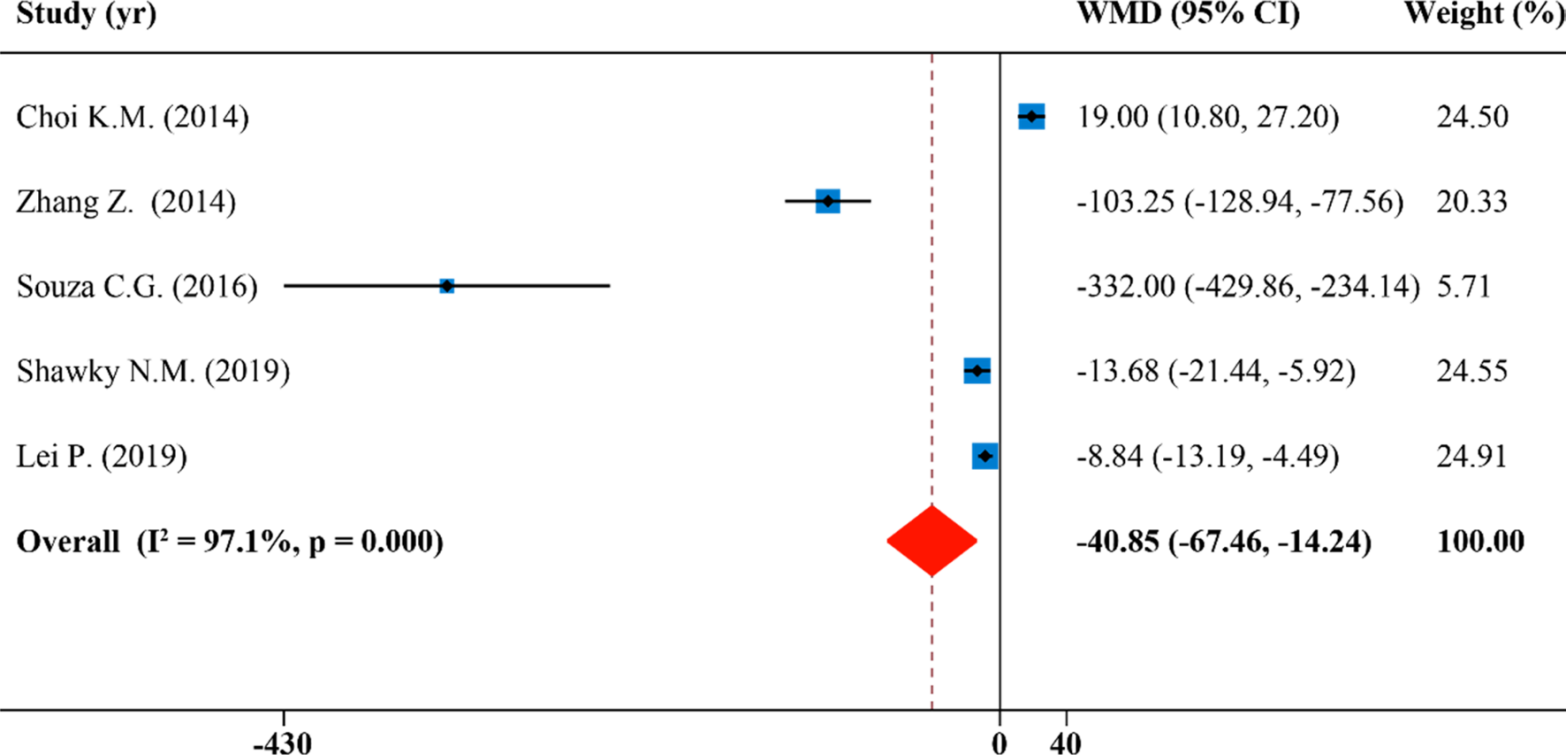
Forest plot showing effects of SFN on serum triglyceride.

There were five effect sizes from 5 publications that were included in the analysis of serum triglyceride. Overall, levels of serum TG were reduced after supplementation of SFN with high degree of study heterogeneity showed by the quantitative meta-analysis (WMD: − 40.85 mg/dL, 95% CI: − 67.46, − 14.24; P = 0.000, I^2^ = 97.1%) (Fig. 7). The results of the subgroup analysis revealed that the heterogeneity among studies could not be explained when studies were divided by species, disease model, duration and SFN dosage and administration route. In these results, the effect of reducing serum TG concentrations after intake of SFN was significant when studies were performed on rodents with a dosage of SFN ≤ 0.5 mg/kg/day (WMD: − 132.41 mg/dL, 95% CI: − 231.71, − 33.12; P = 0.000, I^2^ = 97.5%), with an intervention duration of > 10 weeks (WMD: − 103.25 mg/dL, 95% CI: − 128.94, − 77.56), and using a rat group (WMD: − 33.14 mg/dL, 95% CI: − 60.77, − 5.51; P = 0.000, I^2^ = 95.3%) . In addition, SFN supplements were not statistically significant for other subgroups (Table 2).

### Publication bias and sensitivity analysis

There was no evidence for publication bias through Funnel plots and Egger’s tests (BW Egger’s test: P = 0.201, LW Egger’s test: P = 0.386, TC Egger’s test: P = 0.055, LDL-C Egger’s test: P = 0.515, HDL-C Egger’s test: P = 0.836 and TG Egger’s test: P = 0.230) (Supplementary Table S1 and Figure S1). Sensitivity analyses revealed that any individual study did not influence the summary effects on BW, LW, TC, LDL-C, HDL-C and TG (Supplementary Figure S2).

## Discussion

In this updated meta-analysis, ten articles were utilized to assess SFN supplementation effects on body weight and lipid profile in preclinical animal models. Our analysis clearly demonstrates that SFN supplementation significantly decreased BW, LW, TC as well as LDL-C levels, apart from HDL-C. This is the first meta-analytic study that summarizes the function of SFN mono-treatment on lipid profile in rodents with metabolic syndrome. Clearly, SFN has a positive effect in reducing BW and LW and promotes physiologically lipid profile in animal models.

Our results reveal that different disease models (with or without STZ) do not influence the previous results of meta-analysis on lipid-related parameters and weight in rodents after supplementation with SFN. Duration, dosage of SFN or route of administration has no effect on this result.

Our research focuses on the effects of SFN as a mono-therapeutic drug on animal lipid profile. Majority clinical trials used of broccoli and broccoli sprouts instead of SFN mono-treatment. However, clinical trials with intake of broccoli (enriched with SFN) can provide some auxiliary support for SFN mono-therapeutic research. Adriana Conzatti and coworkers’ study revealed that broccoli sprouts could improve lipid profile and blood gulcose^13^. Armah et al.^17^, also found that plasma LDL-C was significantly downregulated with intake of high glucoraphanin broccoli. However, according to the result of Sudini et al.^39^, intake of broccoli sprouts lasting for half a week did not ameliorate inflammation and oxidative stress markers, in spite of causing a remarkable increase in serum SFN levels. Overall, all related clinical trials we mentioned here, using food like broccoli instead of SFN mono-treatment as therapeutic intervention, required longer time courses to obtained reliable physiologically effects.

In vivo and in vitro studies had shown that SFN can improve lipid-related metabolic indicators and ameliorate cardiovascular disorders^40,41^. Recently, SFN was reported to attenuate HFD-induced obesity through inhibiting lipogenesis via AMP-activated protein kinase (AMPK) pathway^20,29^. SFN played a positive role in cardiomyopathy that was specifically related to the Nrf2-mediated antioxidant pathways (nuclear factor, erythroid 2 like 2) and the AMPK-upregulated lipid metabolism^29,42,43^. Clinical and animal studies shows that the preventive effect of SFN on CVD could also be through Nrf2 activation^44^. Apart from mediating metabolic syndromes, SFN was also found to reduce glycated hemoglobin and fasting blood glucose in type 2 diabetes patients^14,42,45^. Fu et al.^46^ showed that SFN supplements attenuated reactive oxygen species stimulated by glucose, and thereby decreased insulin secretion. SFN could ameliorate obesity and insulin resistance in parallel experiments^31,47–49^.

Clearly, the take home message is that SFN could attenuate certain risk factors of metabolic syndrome through weight management and reduction of lipid abnormalities^12,50^. This is the first systematic review and meta-analysis of SFN monotreatment *vs.* whole broccoli on lipid distribution in rodents with metabolic syndrome. Our subgroup analysis focused on model method, age, SFN dosage, intervention duration and route of administration. Furthermore, the meta-analysis involved 10 studies from various countries and animal models.

However, some limitations of this study must be kept in mind. First, it is not clear whether gender responses differently to the effect of SFN, because all researches use male animals rather than female. Female and male animals have different sex hormones, which may affect serum lipid concentration^51^. It’s necessary to conduct research using both male and female to evaluate the effect of SFN on the lipid profile. Moreover, our meta-analysis is based on animal experiments rather than RCTs. Results obtained through animal models are not necessarily applicable to humans. In addition, only ten studies meet our requirements. The number of studies with clearly delineated data about metabolic parameters is too small to engage in further subgroup analysis. Most articles were not exclusively performed on rodents fed with HFD and the trail using other food supplements with SFN was not utilized here owning to exclusion criterion.

Overall, our analysis supports the conclusion that SFN supplements decrease the level of BW, LW and lipid profile such as TC, TG, LDL-C in rodents. However, this needs to be validated by relevant clinical trials. In addition, it will be necessary to design and perform a more comprehensive panel of indicators in patients with conditions including dyslipidemia, obesity, CVD, NAFLD and related metabolic disorders.

## Supplementary Information

Supplementary Information.
