# Supplementary material for: Sulforaphane ameliorates lipid profile in rodents: an updated systematic review and meta-analysis
Source: Sci Rep. 2021 Apr 8;11:7804. doi: 10.1038/s41598-021-87367-9 (PMC8032686; doi:10.1038/s41598-021-87367-9)

**Supplementary Information**

**Title:** Sulforaphane ameliorates lipid profile in rodents: An updated systematic review and meta-analysis

**Author list:** Kaili Du^1^, Yuxin Fan^1^ and Dan Li^1, #^

^1^Collaborative Innovation Center of Yangtze River Delta Region Green Pharmaceuticals, College of Pharmaceutical Sciences, Zhejiang University of Technology, Hangzhou, China.

# Correspondence: D. L. (Email: [**lidan@zjut.edu.cn**](mailto:lidan@zjut.edu.cn))

**Supplementary Table S1.** Publication bias examined by Egger’s linear regression test.

|  | BW | LW | TC | LDL-C | HDL-C | TG |
| --- | --- | --- | --- | --- | --- | --- |
| Egger's test | 0.201 | 0.386 | 0.055 | 0.515 | 0.836 | 0.230 |

**Supplementary Figure S1. A.** Funnel plots for body weight analysis. **B.** Funnel plots for liver weight analysis. **C.** Funnel plots for serum total cholesterol analysis. **D.** Funnel plots for serum low-density lipoprotein cholesterol analysis. **E.** Funnel plots for serum high-density lipoprotein cholesterol analysis. **F.** Funnel plots for serum triglyceride analysis.

A


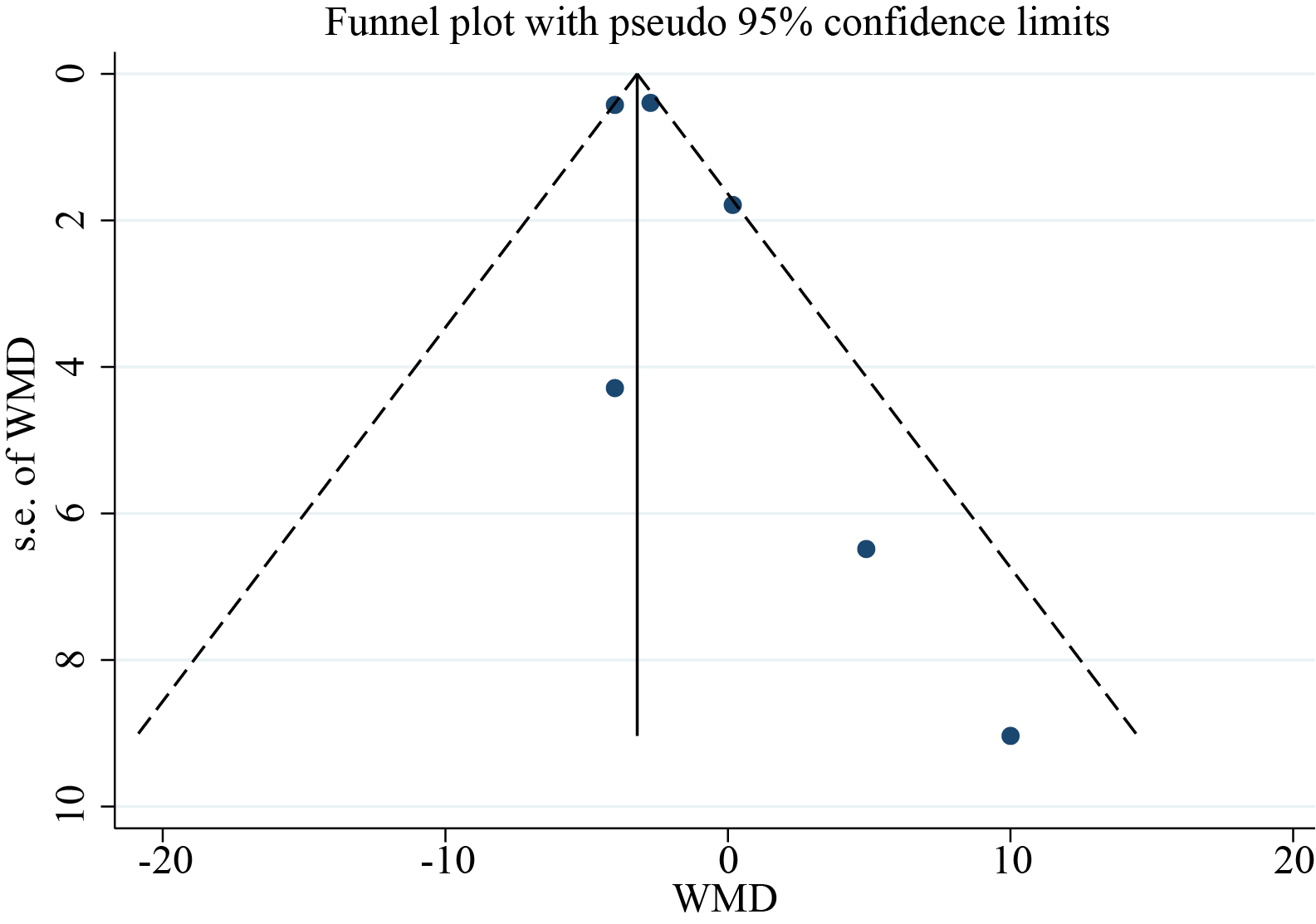


B


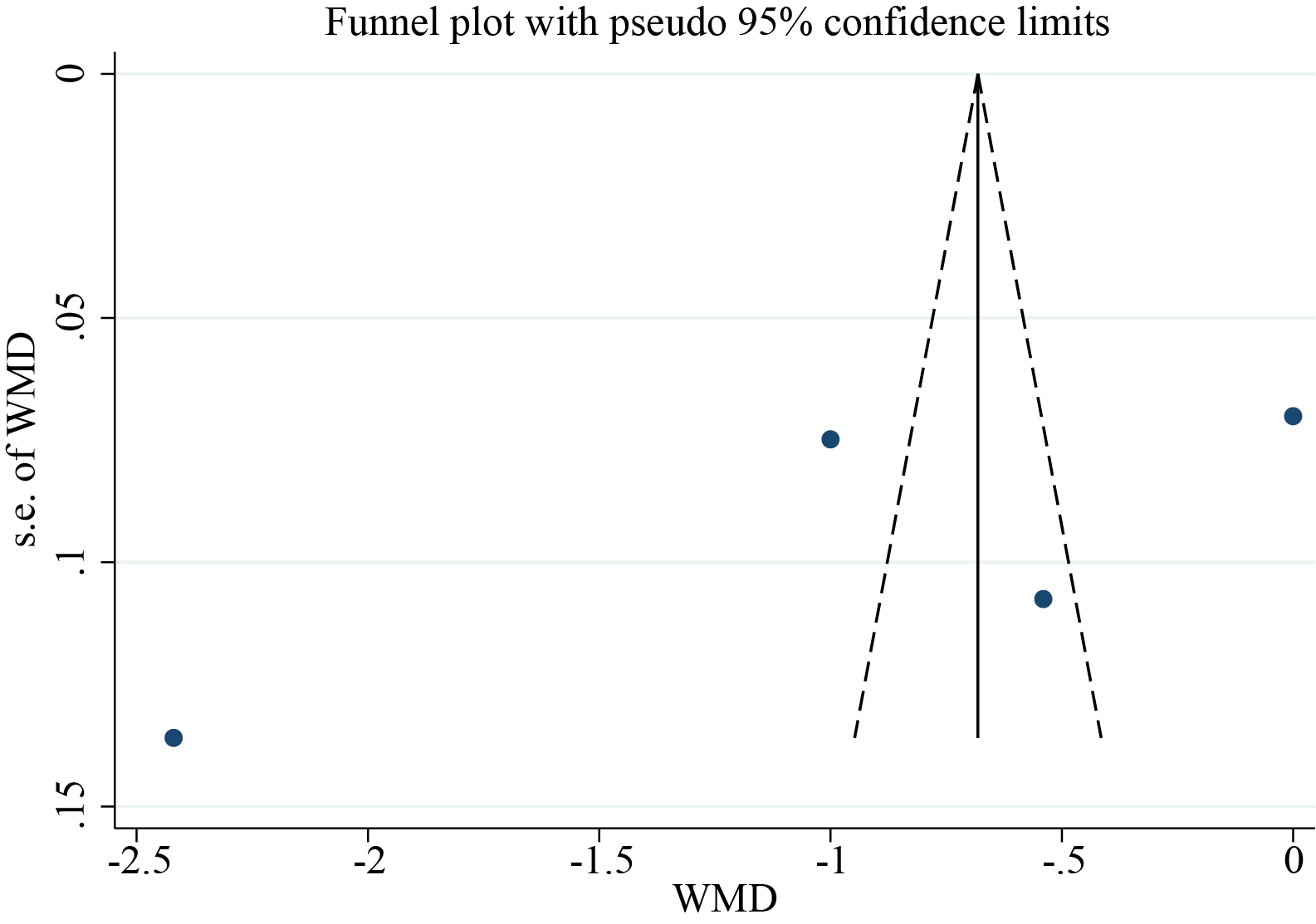
­­

C


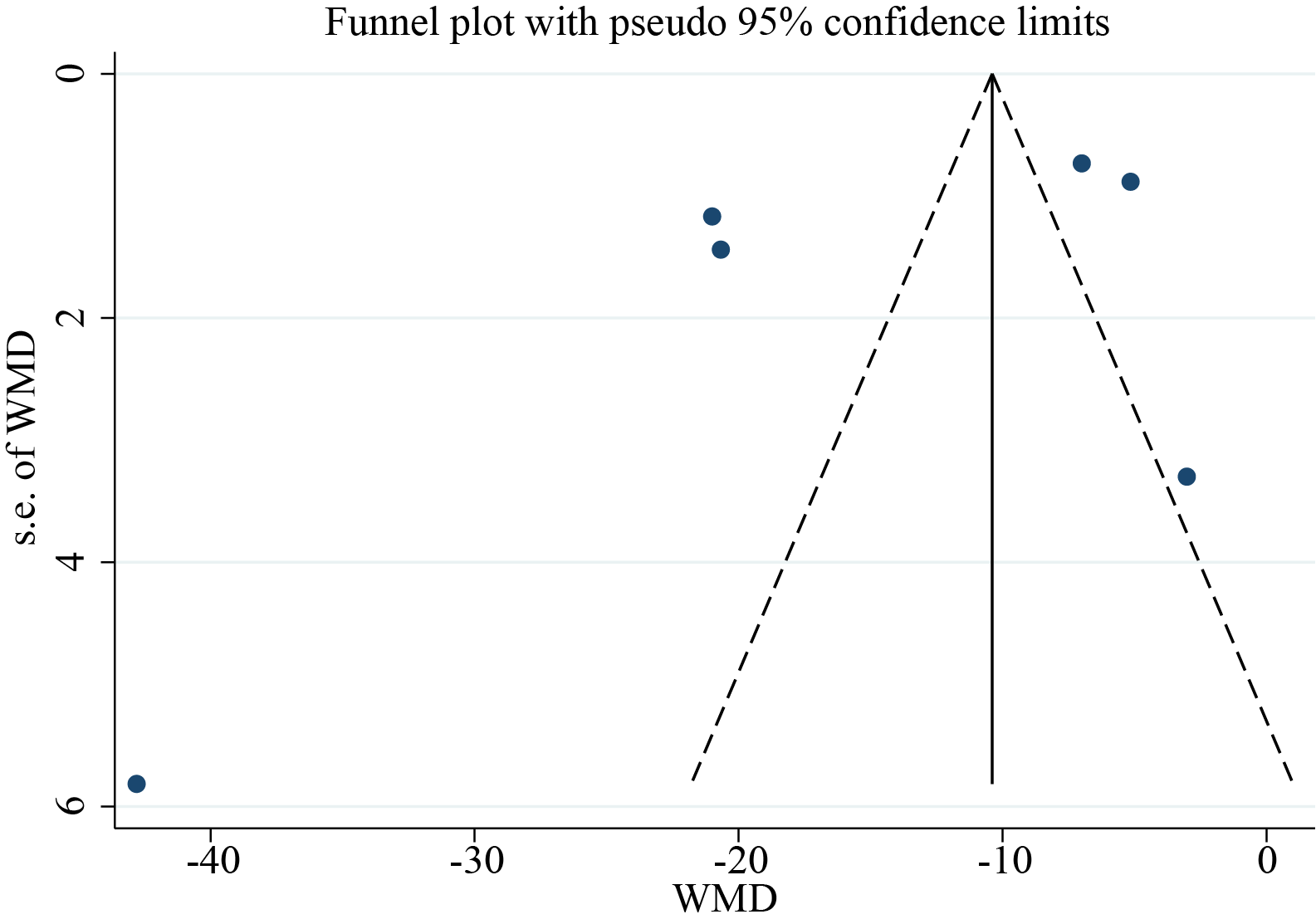


D


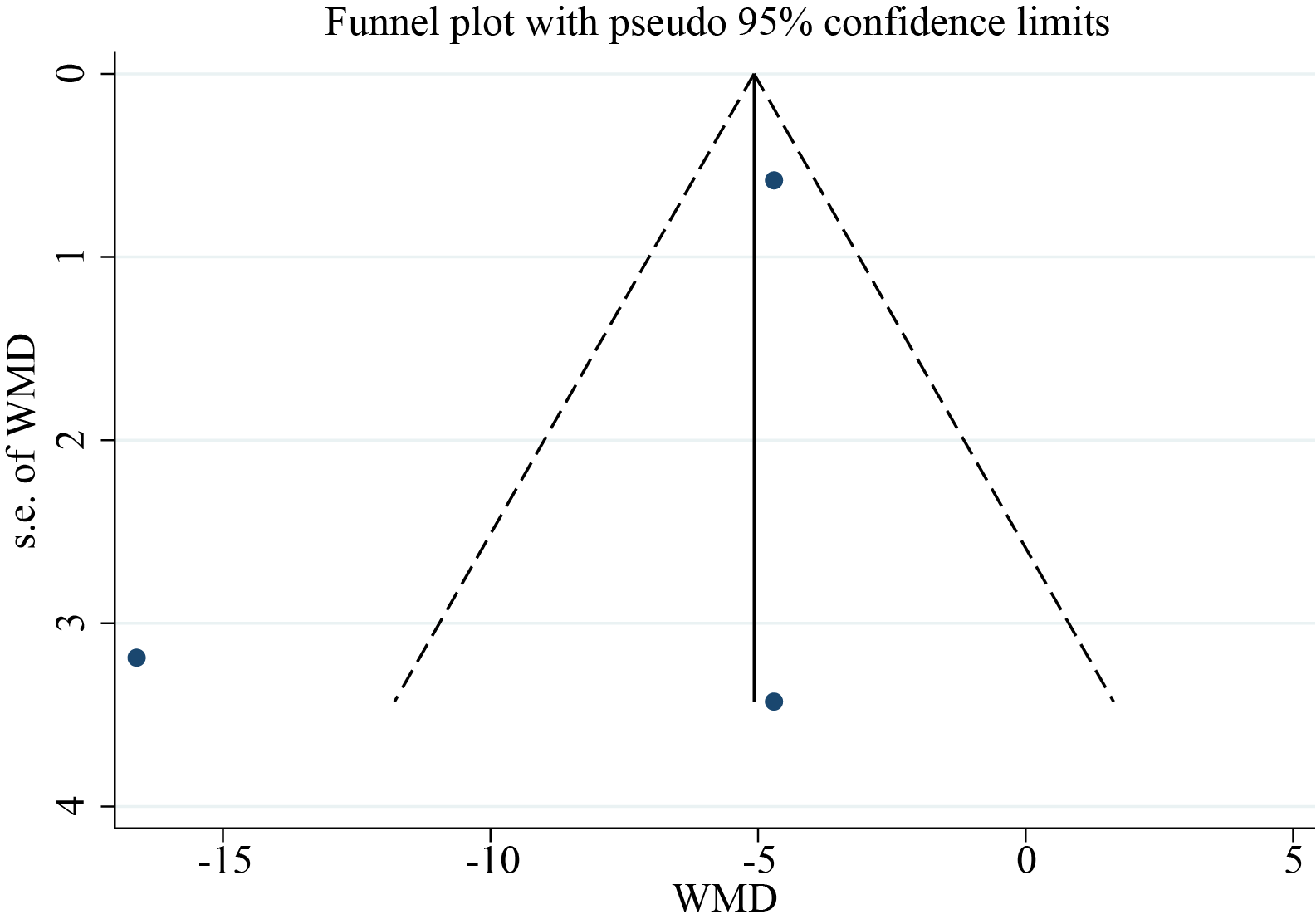


E


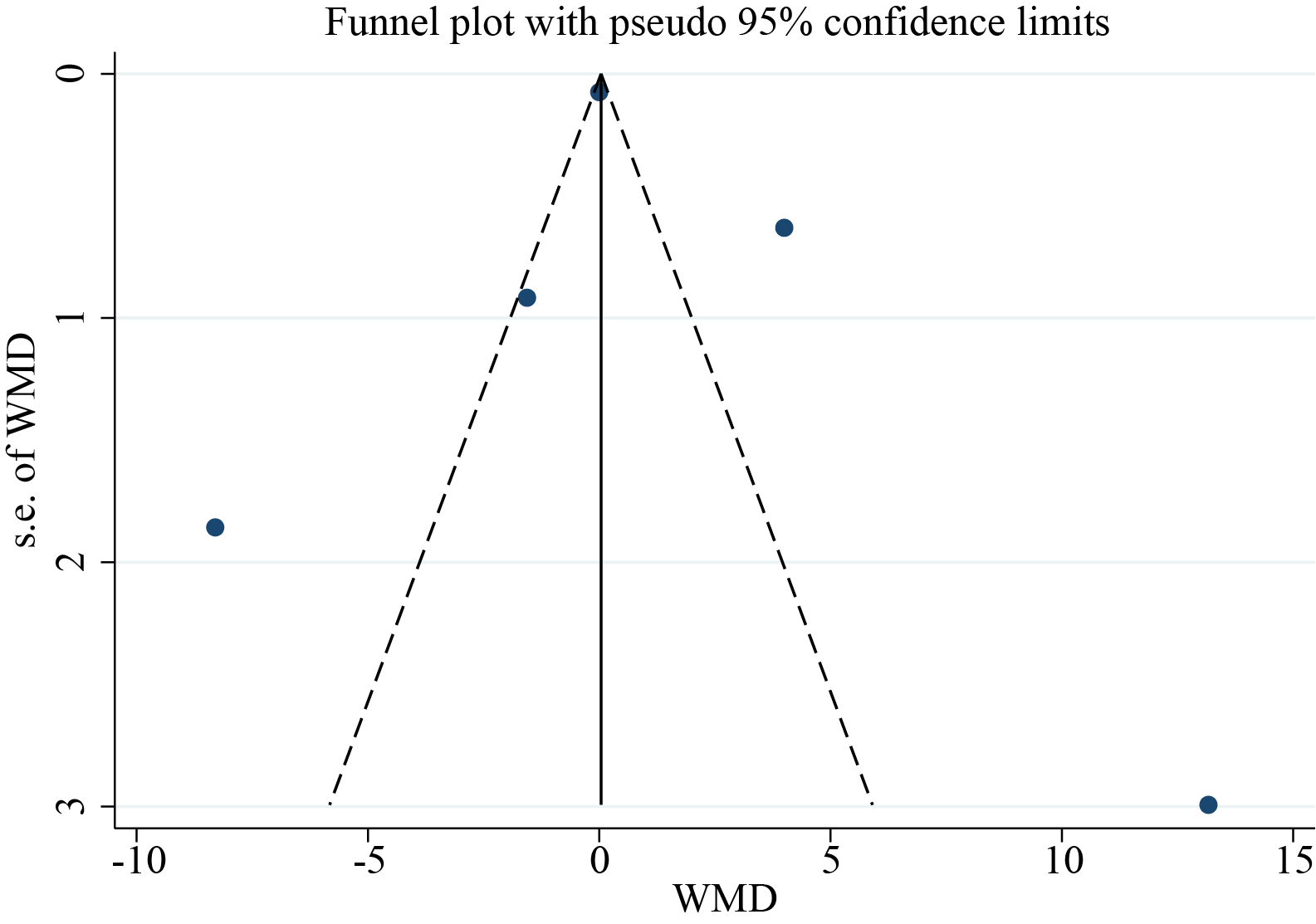


F


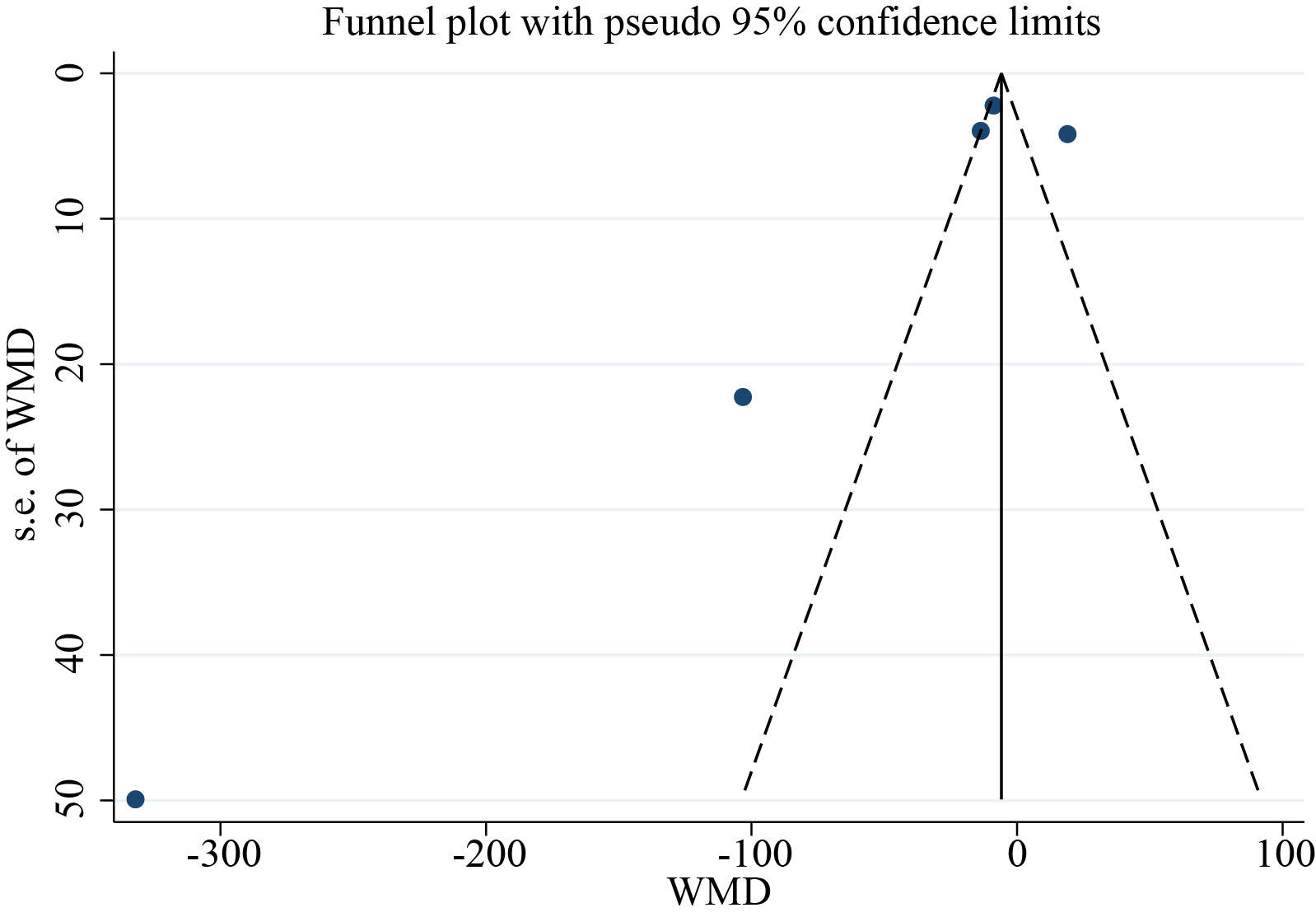


**Supplementary Figure S2. A.** Sensitivity analysis for the association between SFN supplement and body weight. **B.** Sensitivity analysis for the association between SFN supplement and liver weight. **C.** Sensitivity analysis for the association between SFN supplement and serum total cholesterol. **D.** Sensitivity analysis for the association between SFN supplement and serum low-density lipoprotein cholesterol levels. **E.** Sensitivity analysis for the association between SFN supplement and serum high-density lipoprotein cholesterol levels. **F.** Sensitivity analysis for the association between SFN supplement and serum triglyceride.

A


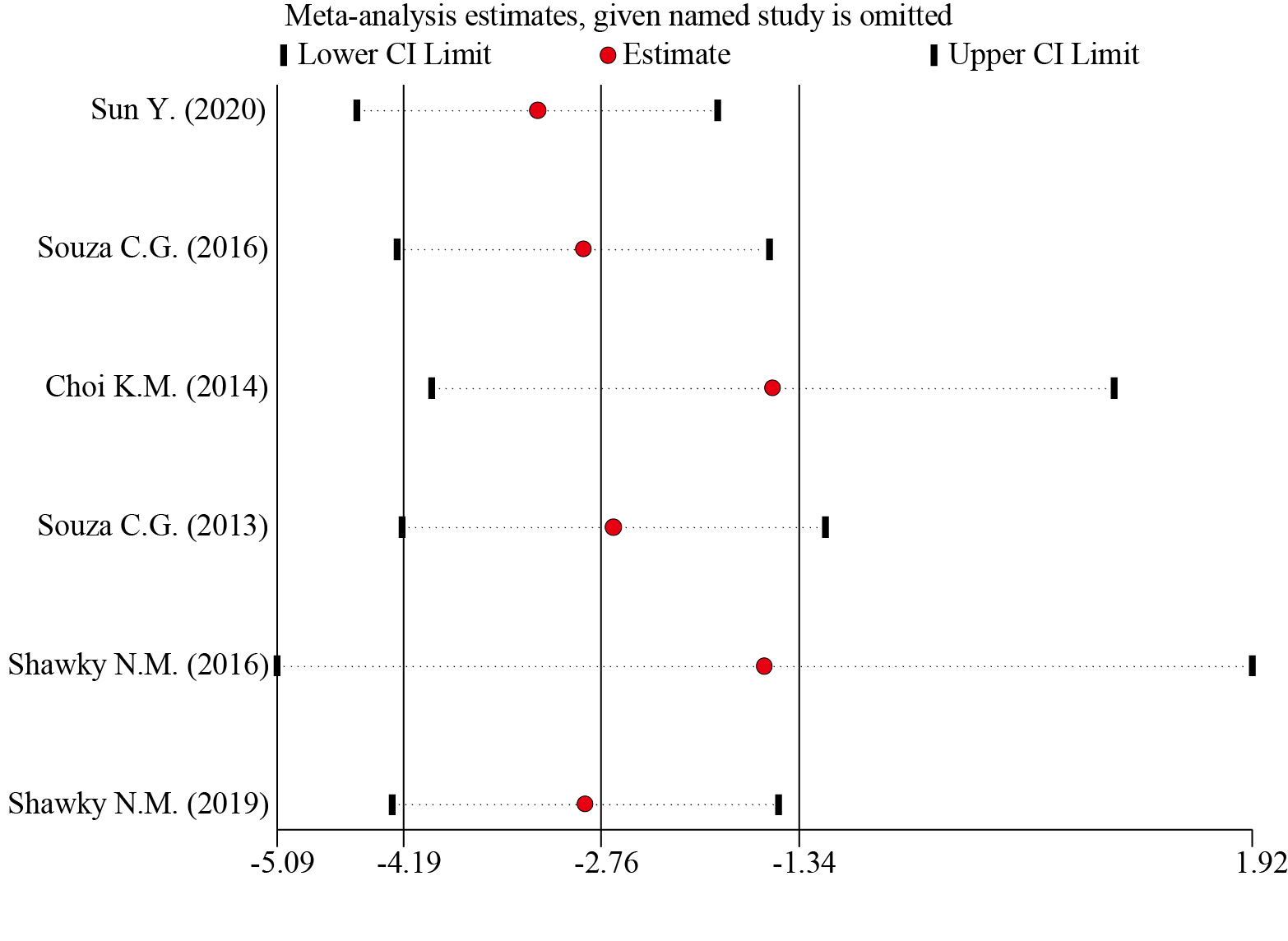


B


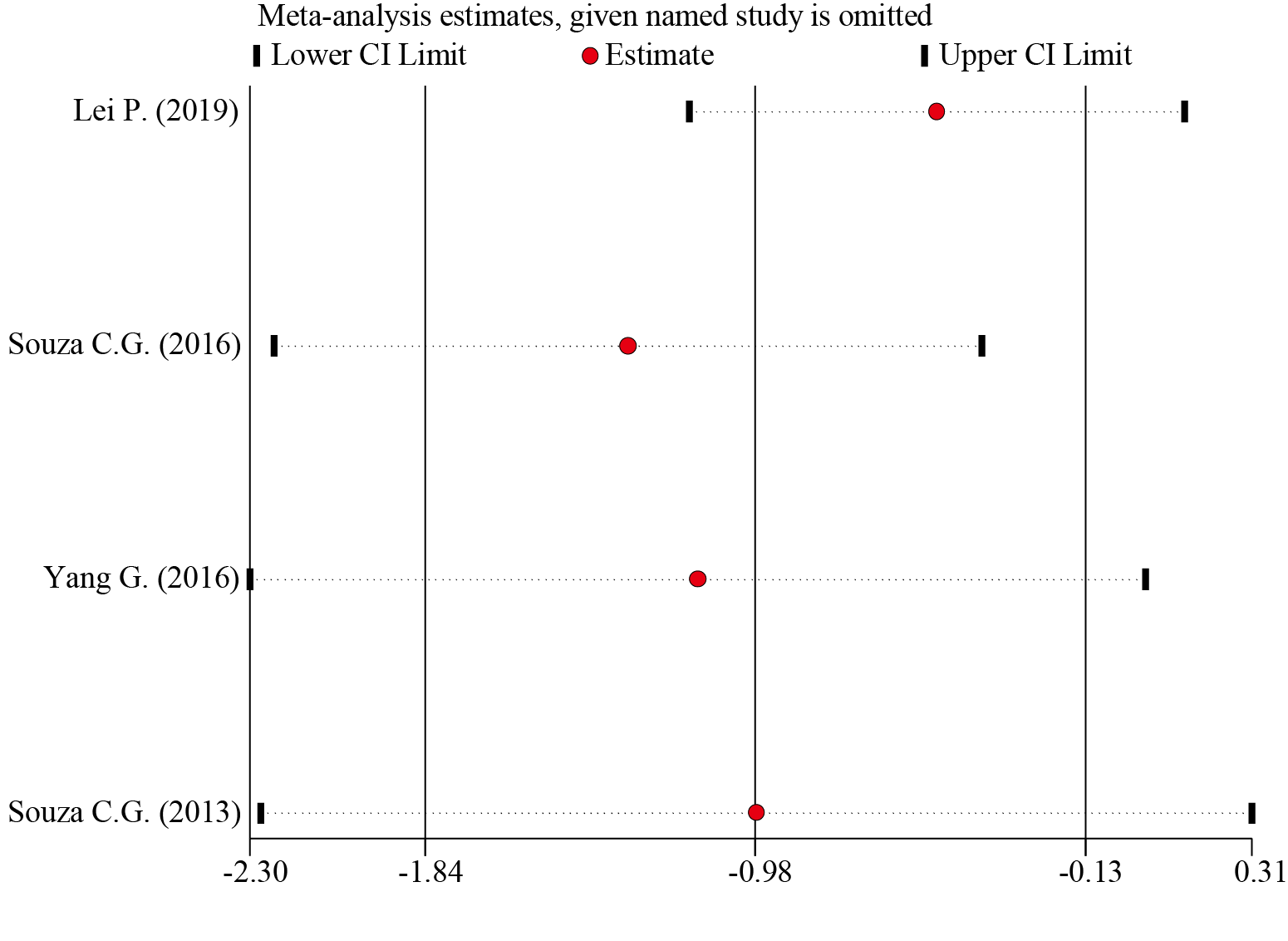


C


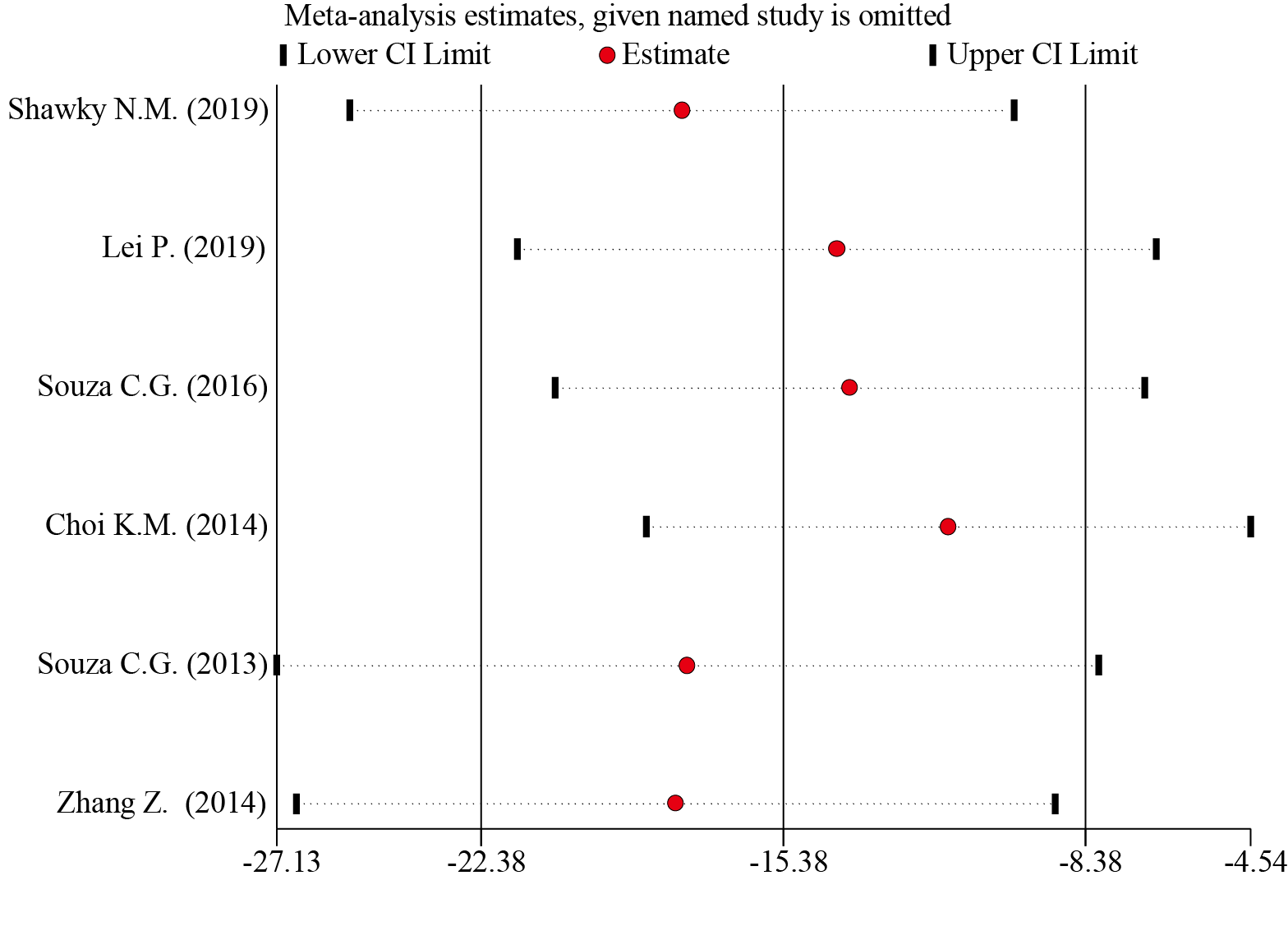


D


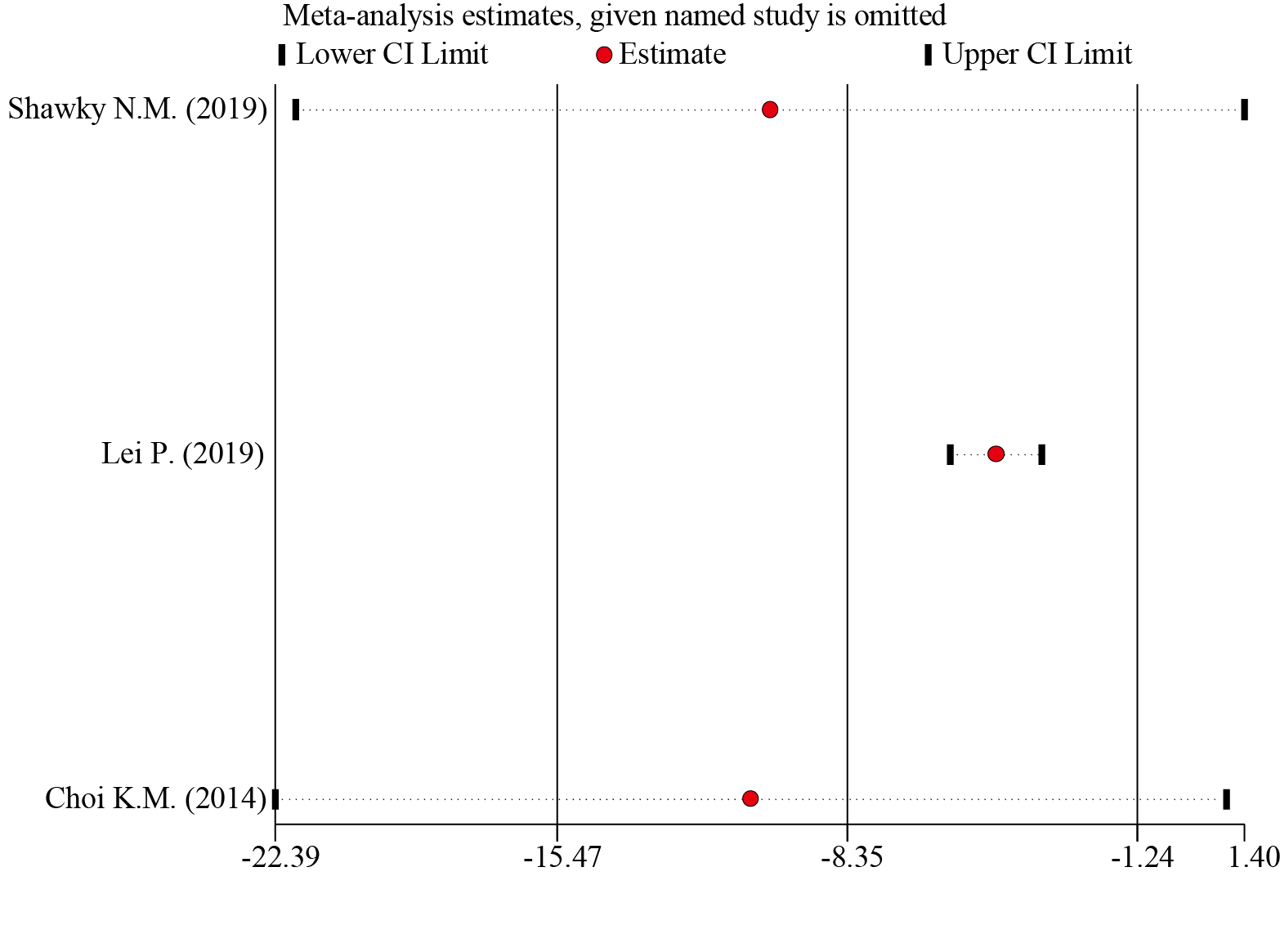


E


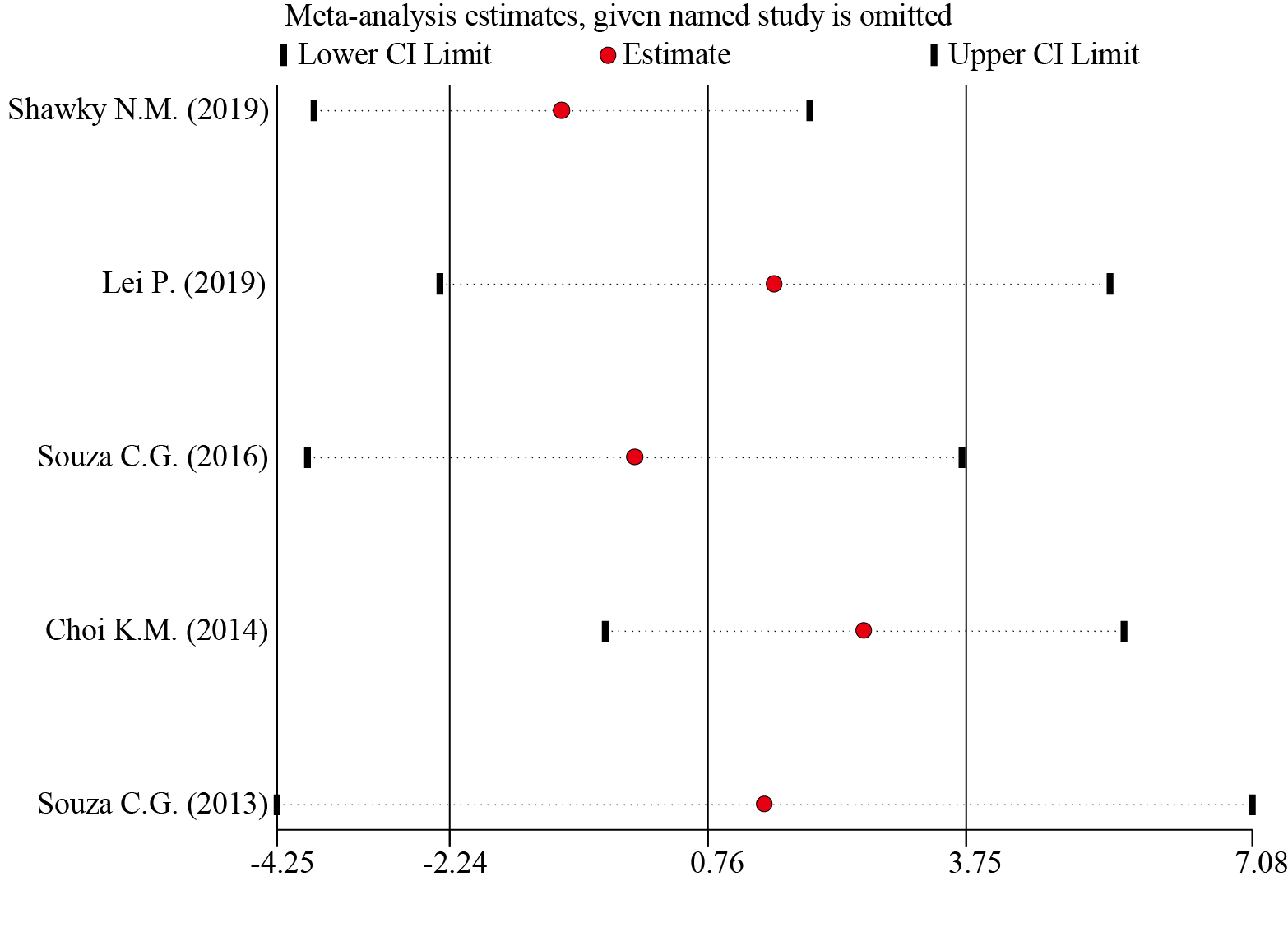


F


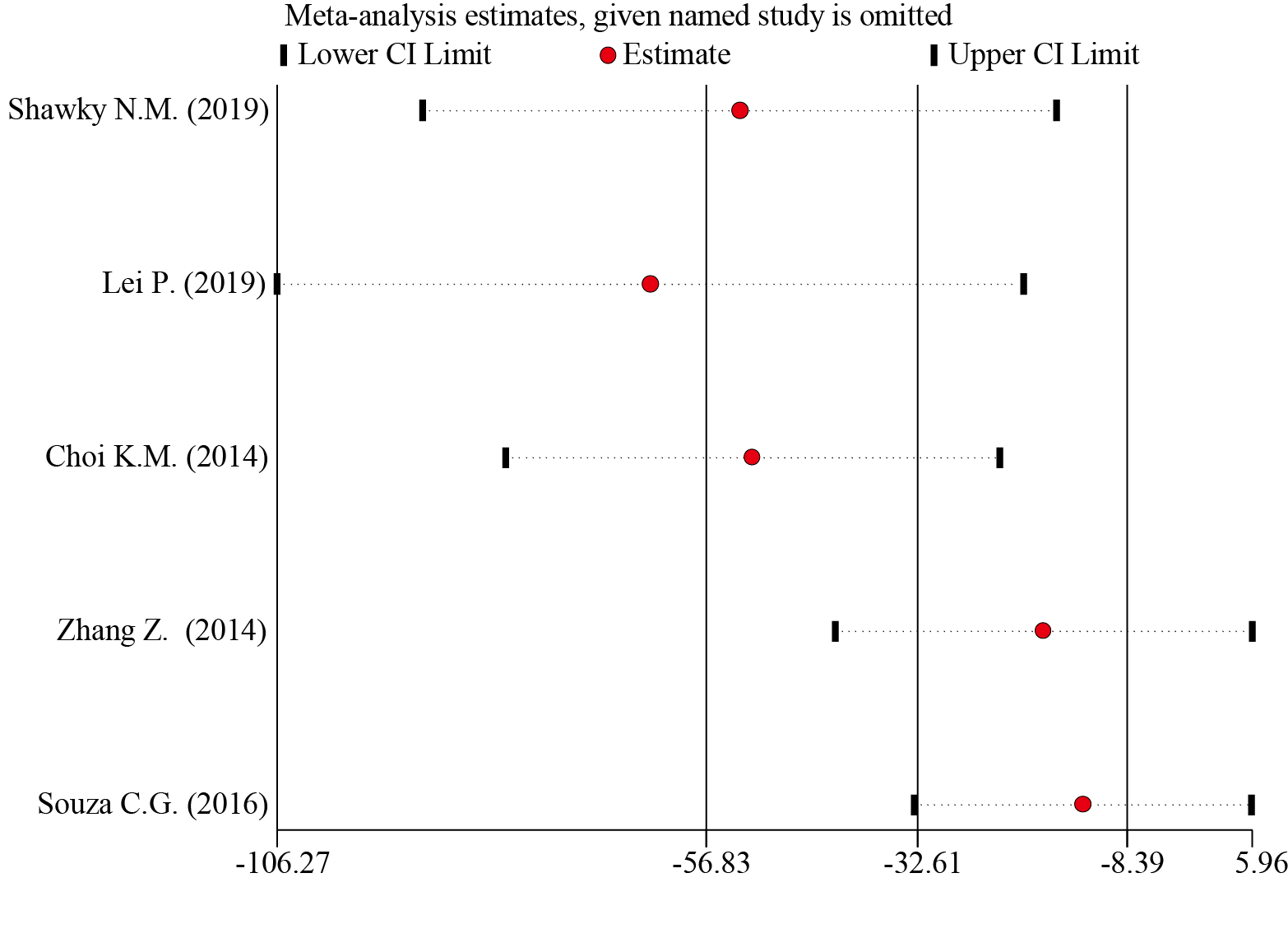

Supplement: Supplementary file 1 — Supplementary Information. [file 41598_2021_87367_MOESM1_ESM.docx]
